# Comparative genomics reveals putative evidence for high-elevation adaptation in the American pika (*Ochotona princeps*)

**DOI:** 10.1093/g3journal/jkac241

**Published:** 2022-09-10

**Authors:** Bryson M F Sjodin, Michael A Russello

**Affiliations:** Department of Biology, University of British Columbia, Okanagan Campus, Kelowna, V1V 1V7 BC, Canada; Department of Biology, University of British Columbia, Okanagan Campus, Kelowna, V1V 1V7 BC, Canada

**Keywords:** comparative genomics, local adaptation, cold tolerance, UV radiation, hypoxia, Lagomorpha

## Abstract

High-elevation environments have lower atmospheric oxygen content, reduced temperatures, and higher levels of UV radiation than found at lower elevations. As such, species living at high elevations must overcome these challenges to survive, grow, and reproduce. American pikas (*Ochotona princeps*) are alpine lagomorphs that are habitat specialists typically found at elevations >2,000 m. Previous research has shown putative evidence for high-elevation adaptation; however, investigations to date have been limited to a fraction of the genome. Here, we took a comparative genomics approach to identify putative regions under selection using a chromosomal reference genome assembly for the American pika relative to 8 other mammalian species targeted based on phylogenetic relatedness and (dis)similarity in ecology. We first identified orthologous gene groups across species and then extracted groups containing only American pika genes as well as unclustered pika genes to inform functional enrichment analyses; among these, we found 141 enriched terms with many related to hypoxia, metabolism, mitochondrial function/development, and DNA repair. We identified 15 significantly expanded gene families within the American pika across all orthologous gene groups that displayed functionally enriched terms associated with hypoxia adaptation. We further detected 196 positively selected genes, 41 of which have been associated with putative adaptation to hypoxia, cold tolerance, and response to UV following a literature review. In particular, *OXNAD1*, *NRDC*, and those genes critical in DNA repair represent important targets for future research to examine their functional implications in the American pika, especially as they may relate to adaptation to rapidly changing environments.

## Introduction

The environment in which a species resides can have a profound impact on its evolution (Parsons 2005; Kristensen *et al.* 2020). High-elevation environments offer a unique combination of challenges that can influence natural selection; these include lower atmospheric oxygen (i.e. hypoxia), reduced ambient temperatures, and increased exposure to DNA-damaging UV radiation relative to lower elevations (Sun *et al.* 2018). The adaptations to these abiotic factors have been shown in a multitude of species. For example, Qiu *et al.* (2012) compared the domestic yak (*Bos grunniens*) genome to that of low-altitude cattle (*Bos taurus*) and found evidence for functional enrichment in energy metabolism and domains related to hypoxic stress. A comparative genomic investigation coupled with functional assays of Tibetan hot-spring snakes (*Thermophis* spp.) identified specific amino acid substitutions for proteins involved in DNA damage repair and response to hypoxia among the high-elevation species (Li *et al.* 2018). Likewise, a transcriptomic analysis of lizards (*Phrynocephalus erythrurus*) on the Qinghai-Tibetan Plateau (QTP) revealed putative adaptations related to hypoxia, energy metabolism, and responses to UV damage (Yang *et al.* 2015). Genome-enabled research into high-elevation systems continues to expand our knowledge of adaptation to extreme environments.

The American pika (*Ochotona princeps*) is an alpine lagomorph distributed across mountain ranges in the Pacific Northwest (Smith and Weston 1990; Hafner and Smith 2010). They are considered habitat specialists and typically reside in rocky, talus slopes at elevations >2,000 m (Smith and Weston 1990; Millar and Westfall 2010; Smith and Beever 2016). Previous research has provided some evidence for American pika high-elevation adaptation on local/regional scales based on reduced representation genome sequencing (Waterhouse *et al.* 2018; Schmidt *et al.* 2021) and transcriptomics (Lemay *et al.* 2013); however, a comprehensive examination across the American pika genome has not been explored. Furthermore, previous work relied upon older, lower-quality reference assemblies for the American pika; for example, Wang *et al.* (2020) used OchPri2.0-Ens (Ensembl), which is highly fragmented (193,096 scaffolds; scaffold N50 = 53.58 kb) and poorly annotated (16,006 genes) relative to modern assemblies (Giani *et al.* 2020; Whibley *et al.* 2021). The most recent reference genome for the American pika (OchPri4.0; NCBI RefSeq Accession: GCF_014633375.1) is significantly improved in both contiguity (9,350 scaffolds; scaffold N50 = 75.8 Mb) and annotation quality (21,186 genes), and is almost entirely (97% of total length) assembled into chromosomes (Sjodin *et al.* 2021). Importantly, the American pika has emerged as a sentinel species for the ecological impacts of climate change in alpine regions due to their acute environmental sensitivity (Beever *et al.* 2003; Galbreath *et al.* 2009; Beever *et al.* 2010, 2011; Wilkening *et al.* 2011; Stewart *et al.* 2015; Schwalm *et al.* 2016; Wilkening and Ray 2016); a thorough examination of climate adaptation within the American pika genome may provide important insights into potential biotic responses to changing environments while representing a valuable resource for guiding future studies.

Here, we investigated signatures of putative adaptation to high-elevation environments in the American pika. Leveraging the newest version of the *O. princeps* genome (Sjodin *et al.* 2021), we compared orthologous genes among the American pika and 8 other mammalian species to estimate phylogenetic relationships, examine functional enrichment in the American pika genome, and detect signatures of positive selection. We then identified putative genomic adaptations to high-elevation environments based on characterized gene functions and a targeted literature review.

## Methods and materials

### Study design

We leveraged the recently published and annotated American pika genome (Sjodin *et al.* 2021) to identify putative genomic adaptations to high-elevation environments by comparing and contrasting with 8 other paired mammalian species spanning several major taxa across Glires (i.e. lagomorphs and rodents). Each species pair included a habitat generalist and an alpine habitat specialist to also cover a broad ecological range. The habitat specialists were the American pika, long-tailed chinchilla (*Chinchilla lanigera*), alpine marmot (*Marmota marmota marmota*), and arctic ground squirrel (*Urocitellus parryii*), whereas the paired generalists were European rabbit (*Oryctolagus cuniculus*), common degu (*Octodon degus*), yellow-bellied marmot (*Marmota flaviventris*), and 13-lined ground squirrel (*Ictidomys tridecemlineatus*), respectively. Humans (*Homo sapiens*) were included as an outgroup. To minimize differences in annotation quality across genomes, we used only those available through the NCBI RefSeq database and annotated using the NCBI Eukaryotic Genome Annotation Pipeline (O’Leary *et al.* 2016). Coding domain sequences (CDSs) and protein FASTA files were downloaded for each species from NCBI and used for downstream analyses (see Supplementary Table 1 for accession information).

### Detection of orthologous gene families and functional enrichment

American pika genes were aligned and functionally annotated against the nonredundant NCBI and SwissProt (Boeckmann *et al.* 2003) protein databases using BLASTP *v*2.9.0 (Camacho *et al.* 2009) with an *E*-value cut-off of 1e−5. Genes with BLAST hits were then annotated with gene ontology (GO) terms using Blast2GO *v*5.2.5 (Götz *et al.* 2008). In addition, American pika genes were grouped into protein families and annotated with GO terms using InterProScan *v*5.50-84.0 (Cock *et al.* 2013; Jones *et al.* 2014) under default settings and merged with the BLAST GO terms in Blast2GO *v*5.2.5 (Götz *et al.* 2008).

We then identified orthologous gene families between the American pika and the other 8 mammalian species. To reduce redundancy within the dataset, we retained only the longest isoform for each protein within a species; in addition, we removed proteins with fewer than 50 amino acids to minimize false positives during ortholog detection and clustering. Orthologous protein sequences both within and among species were identified first using reciprocal best BLAST hits then clustered into gene families using a Markov Clustering algorithm as implemented in a modified version of OrthoMCL *v*2.0.9 (Fischer *et al.*, 2011; www.github.com/apetkau/orthomclsoftware-custom). Ortholog detection was automated with the orthomcl-pipeline using default settings (www.github.com/apetkau/orthomcl-pipeline).

We extracted gene families containing only American pika genes and grouped these with unclustered American pika genes to construct a “pika-specific” gene set. We found functionally enriched GO terms among pika-specific genes by means of a hypergeometric test using BiNGO *v*3.0.4 (Maere *et al.* 2005) as implemented in Cytoscape *v*3.8.2 (Smoot *et al.* 2011) employing the entire American pika gene set as the reference. Obtained *P*-values were corrected for multiple testing using the Benjamini–Hochberg false discovery rate, and significantly enriched GO terms were identified with an adjusted *P ≤ *0.05. GO terms with similar functions were grouped together based on quantified information content and semantic similarities as calculated in the software GO-Figure! *v*1.0.1 (Reijnders and Waterhouse 2021).

### Phylogenetic reconstruction and estimation of divergence times

To generate an ultrametric tree for downstream analysis, single-copy orthologs were extracted from the orthologous gene families, defined as those families with a single representative gene from each species. We removed gene families with any sequences shorter than 200 amino acids to minimize spurious alignments and conducted a multiple sequence alignment using MUSCLE *v*3.8.31 (Edgar 2004a, 2004b) and default parameters. The corresponding CDS alignments were back-translated using PAL2NAL *v*14 (Suyama *et al.* 2006). Some CDSs did not contain chromosomal locations as they were predicted based on transcriptomic or other sequencing data in the RefSeq database; gene families containing these unlocalized genes were removed from downstream analysis. We identified homologous gene blocks for the remaining orthologous groups and concatenated them into “supergenes” using Gblocks *v*0.91b (Talavera and Castresana 2007). We then identified and extracted 4-fold degenerate nucleotide sites (4DTV) using MEGA *v*10.2.5 (Tamura *et al.* 2021) and used these sites to reconstruct the phylogeny under the GTR+I+Γ model as implemented in MrBayes *v*3.2.6 (Ronquist *et al.* 2012). We ran 5 independent runs of the Markov chain Monte-Carlo (MCMC) process for 5 million generations with a 1 million generation burn-in each, sampling trees every 100 generations. Convergence was assessed by examining the estimated sample size (ESS) and potential scale reduction factor (PSRF) for each parameter estimate. Convergence was achieved when ESS > 100 and PSRF ≈ 1 for all parameters (see Supplementary Table 2 for summary values).

To estimate divergence times, we first separated the concatenated super genes into 3 datasets corresponding to the first, second, and third codon sites. Divergence times were then estimated under a relaxed clock model using MCMCTREE as implemented in PAML *v*4.9 (Yang 2007). The tree topology was defined using the outputs from the above analysis. The mean substitution rate was estimated using BaseML in PAML *v*4.9 (Yang 2007). The overall substitution rate prior (rgene_gamma) was set to [1, 8] and the rate drift parameter prior (sigma2_gamma) was set to [1, 10, 1] following author recommendations (Yang 2007). We used 3 calibration points based on previous studies: the split between Primates and Glires constrained at 61.7–100.5 million years ago (mya; Benton and Donoghue 2007); the split between Rodentia and Lagomorpha constrained at 71.5–94.1 mya (Meredith *et al.* 2011); and the split between Leporidae and Ochotonidae constrained at 47.4–56.9 mya (Meredith *et al.* 2011). We ran the program using 6 million MCMC reps and a burn-in of 2 million iterations. Divergence estimates from 2 independent runs were compared to assess convergence. The topology and divergence estimates were compared with established values in the literature to ensure the appropriateness of using this dataset for downstream analyses.

### Identification of expanded and contracted gene families

We identified expanded and contracted gene families along each branch and node from the above phylogeny using CAFE *v*2.0 (Hahn *et al.* 2005, 2007; De Bie *et al.* 2006). We compared the cluster size of each branch with the maximum likelihood cluster size of the ancestral node leading to that branch and identified expanded and contracted gene families as those with smaller or larger ancestral nodes, respectively. We calculated the family-wide *P*-values using a Monte Carlo resampling procedure of each branch and node and calculated the exact *P*-values for each significant family with *P ≤ *0.01 using the Viterbi method in CAFE *v*2.0 (Hahn *et al.* 2005, 2007; De Bie *et al.* 2006). Significant gene family expansions/contractions were defined as those with family *P*-values and exact *P*-values ≤0.01. We extracted genes from significantly expanded American pika gene families and identified functionally enriched GO terms using the methods described above.

### Identification of positively selected genes and putative high-elevation adaptation

We identified positively selected genes (PSGs) in the American pika from the previously identified single-copy orthologs following alignment refinement using Gblocks *v*0.91b (Talavera and Castresana 2007) and a branch-site model using CodeML as implemented in PAML *v*4.9 (Yang 2007); these steps were automated using a custom shell script “p-codeml” (https://github.com/bsjodin/p-codeml). Refined gene alignments with length <150 bp were removed from downstream analysis to minimize spurious results. The American pika was set as the foreground branch, and all other species were set as background branches. We performed a likelihood ratio test, and resultant *P-*values were corrected for multiple testing using an FDR test with a Bonferroni correction. Significant PSGs were defined as those with an adjusted *P ≤ *0.01 and contained at least one positively selected site with a posterior probability ≥0.99 based on Bayes Empirical Bayes (BEB) analysis. We identified functionally enriched GO terms among PSGs using the methods described above. Functional descriptions for all PSGs were automatically generated using the Alliance of Genome Resources website (Kishore *et al.* 2020). To identify PSGs with putative links to high-elevation adaptation, we manually searched each gene on Google Scholar using the following Boolean search term: “gene name” “cold stress” OR “cold response” OR “cold resistance” OR “hypoxia” OR “high altitude” OR “UV damage” OR “climate.” Searches were constrained to the first 10 hits, and putatively high-elevation adaptive genes were identified as those with reference support for adaptation to hypoxia, cold temperatures, and/or UV exposure in any system or organism.

## Results

### Functional enrichment of pika-specific genes

**Fig. 1.**
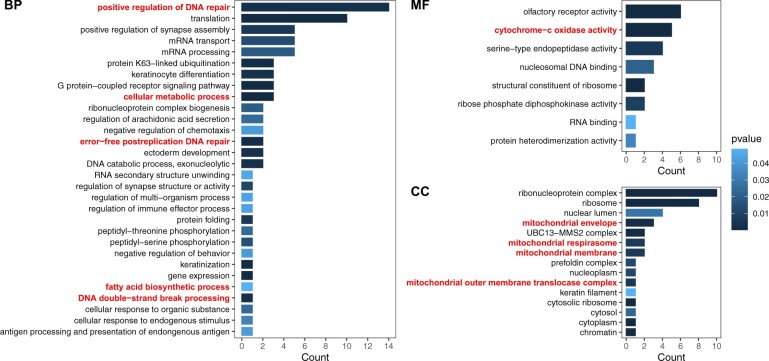
Significantly enriched GO terms for “pika-specific” genes in the American pika genome. Significantly enriched terms were identified using a hypergeometric test using BiNGO *v*3.0.4 (Maere *et al.* 2005) as implemented in Cytoscape *v*3.8.2 (Smoot *et al.* 2011). Semantically similar terms were grouped together using GO-Figure! *v*1.0.1 (Reijnders and Waterhouse 2021). Bolded terms were putatively associated with high-elevation adaptation. BP, biological process; MF, molecular function; CC, cellular component. GO IDs and grouped terms can be found in Supplementary Table 4.

We retained a mean of 20,098 genes from each species after filtering for downstream analysis, including 18,854 genes from the American pika (see Supplementary Table 3). We found 17,127 orthologous gene families across all species; 25 gene families contained only American pika genes (*n *=* *55 genes), and these were grouped with 881 unclustered American pika genes to construct our pika-specific dataset (total *n *=* *936 genes; see Supplementary Table 3). Of these, 857 genes could be annotated with GO terms. We found 141 functionally enriched GO terms (see Supplementary Table 4), which were grouped into 53 parent terms (Fig. 1). Of these parent terms, we identified 10 with putative links to high-elevation adaptation, including: 2 groups with 4 total terms associated with metabolism [cellular metabolic process (GO:0044237) and fatty acid biosynthetic process (GO:0006633)]; 4 groups containing 8 terms enriched in mitochondrial function/structure [mitochondrial envelope (GO:0005740), mitochondrial respirasome (GO:0005746), mitochondrial membrane (GO:0031966), and mitochondrial outer membrane translocase complex (GO:0005742)]; one group with 5 terms related to cytochrome-c oxidase activity (GO:0004129); and 3 groups with a total of 17 terms associated with DNA repair [positive regulation of DNA repair (GO:0045739), error-free post replication DNA repair (GO:0042275), and DNA double-strand break (DSB) processing (GO:0000729)].

### Phylogeny and divergence times from single-copy orthologs

We identified 9,170 single-copy orthologous gene families among all 9 species. Of these, 2,081 groups were removed due to a protein length <50 amino acids and an additional 2,312 groups were removed due to inconsistencies between the protein and CDSs, resulting in a total of 4,777 groups. Gene alignments were refined and concatenated, and 4DTV sites were extracted resulting in 739,038 base “supergenes.”

**Fig. 2.**
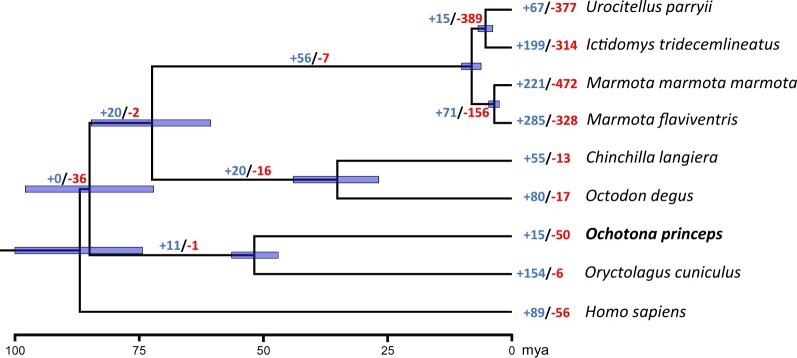
Divergence time estimates and significantly expanded/contracted gene families across 9 mammalian species. Divergence times were estimated under a relaxed clock model as implemented in the MCMCTREE program of PAML *v*4.9 (Yang 2007). Branch labels indicate significantly expanded (+ values) and contracted (- values) gene families and were detected using CAFE *v*2.0 (Hahn *et al.* 2005, 2007; De Bie *et al.* 2006).

Our recovered topology was consistent with the currently recognized phylogenetic relationships (Gupta and Suggett 2022); each recovered node was supported with a posterior probability of 1.0 (Supplementary Fig. 1). In addition, divergence times for all nodes were largely consistent with previous estimates (Fig. 2). Our divergence estimates indicated the split between Primates and Glires occurred ∼87.7 mya (Benton and Donoghue 2007; Meredith *et al.* 2011) with the split between Rodentia and Lagomorpha occurring shortly after at ∼85.7 mya (Benton and Donoghue 2007; Meredith *et al.* 2011). We estimated the split between Sciurimorpha (marmots and ground squirrels) and Hystricomorpha (chinchilla, degu) to have occurred ∼73.0 mya (Montgelard *et al.* 2008). Chinchillidae and Octodontidae diverged next within Rodentia at ∼35.4 mya (Voloch *et al.* 2013). Ground squirrels and marmots diverged much more recently at ∼8.1 mya (Giboulet *et al.* 1997), with within-family divergences occurring at ∼5.3 and ∼3.6 mya, respectively; these latter values were somewhat earlier than previously estimated divergence times using complete cytochrome *b* sequences (Harrison *et al.* 2003). Finally, we estimated that Leporidae and Ochotonidae diverged ∼52.3 mya consistent with previous findings (Wang *et al.* 2020).

### Significantly expanded gene families in the American pika

We found 15 significantly expanded gene families in the American pika genome encompassing 88 genes, of which 83 could be annotated with GO terms (Fig. 2; see Supplementary Table 5). The 15 expanded gene families had functions related to immune response (1 family), transcription/translation (4 families), cell proliferation (1 family), catalytic activity (4 families), olfactory/pheromone receptor activity (3 families), oxidoreductase activity (1 family), and nuclear structural components (1 family). We found 18 significantly enriched GO terms (see Supplementary Table 6 in Supplementary) across these gene families related to translation (3 terms), cellular proliferation (5 terms), immune response (2 terms), membrane receptor activity (2 terms), olfactory/pheromone receptor activity (2 terms), oxidoreductase activity (1 term), catalytic activity (2 terms), and biosynthetic activity (1 term).

### PSGs with putative links to high-elevation adaptation

We removed 7 single-copy orthologs from the previously identified 4,777 due to short length prior to PSG detection. Of the remaining 4,770 genes, we found 196 PSGs within the American pika genome with at least one positively selected site (BEB ≥ 0.99; corrected *P ≤ *0.01; see Supplementary Table 7). One hundred and ninety PSGs were annotated with GO terms; however, we found no significantly enriched GO terms across these genes (see Supplementary Table 8). We identified 41 PSGs with putative implications for adaptation to high-elevation environments, including 16 with putative links to cold tolerance, 23 with links to hypoxia, 7 with links to UV exposure, and another 6 with associations with high-elevation populations in other species (Table 1; *n.b.*, some genes were classified into multiple categories).

**Table 1.** Positively selected genes (PSGs) in the American pika genome with putative links to high-elevation adaptation.

*Gene code*	Adaptation/evidence	References
**Cold**		
*AASS*	Differentially expressed under cold-stress conditions; may affect amino acid content (in winter turnip rape) due to cold stress	Fang *et al.* (2021)
*CAPSL*	Putatively under a selective sweep in mosquitoes in Russia, linked to cold tolerance	Konorov *et al.* (2021)
*DNAJA2*	Upregulated due to cold stress in common carp	Cossins *et al.* (2006)
*DNAJC13*	Involved in cold resistance in Chinese white wax scale insect	Zhang *et al.* (2021)
*EIF4B*	Upregulated due to cold stress in Colorado potato beetle	Govaere *et al.* (2019)
LOC101526896 (*UQCRC2*)	Over-expressed in yaks relative to cattle; improved energy metabolism due to high-altitude adaptation	Wen *et al.* (2019)
LOC101527142 (*CRHBP*)	Increased expression after exposure to cold in Chinese honeybees	Liu *et al.* (2011)
*NRDC*	Critical for thermogenesis and temperature homeostasis in mice/mammals	Hiraoka *et al.* (2014)
*NUP205*	Increased expression following cold stress in large yellow croaker	Qian and Xue (2016)
*PHKB*	Involved in cold acclimation in fish	Healy and Schulte (2019)
*PLA1A*	Linked to SNP outlier in cold-resilient cattle breeds	Igoshin *et al.* (2021); Passamonti *et al.* (2021)
*PSMA6*	Under-expressed following chronic cold stress in gilthead sea bream	Sanahuja *et al.* (2019)
*SUGT1*	Linked to temperature stress in black rockfish	Lyu *et al.* (2018)
*TBCD*	Associated with outlier SNP linked to temperature stress-response in red mullet	Boulanger *et al.* (2022)
*TREH*	Plays a key role in cold resistance across numerous species	Shi *et al.* (2016); Bao *et al.* (2021)
*ZNF330*	Upregulated in response to cold stress in rainbow trout kidney	Verleih *et al.* (2015)
**Hypoxia**		
*AASS*	Upregulated in mice exposed to oxidative stress	Bertoletto *et al.* (2012)
*ACTR2*	Downregulated in human macrophages under chronic hypoxia	Fuhrmann *et al.* (2013)
*ADAL*	Upregulated with chronic hypoxia	Van Linden and Eltzschig (2007)
*BCKDHA*	Upregulated in hypoxic conditions (in bacteria)	Oliveira *et al.* (2021)
*CUL1*	May be linked to hypoxia	Mikus and Zundel (2005)
*EIF4B*	Increased phosphorylation in liver of naked mole rats following hypoxia	Al-attar *et al.* (2020)
*EIF5B*	Upregulated in response to hypoxia in primary endothelial cells	Bartoszewski *et al.* (2019)
*GBE1*	Upregulated in hypoxic conditions	Pescador *et al.* (2010); Goodin *et al.* (2013)
*HAUS3*	Downregulated under hypoxia in threespine stickleback	Leveelahti *et al.* (2011)
LOC101527142 (*CRHBP*)	Upregulated in hypoxic conditions for developing rainbow trout	Fuzzen *et al.* (2011)
*MAT2B*	Downregulated under hypoxia in common sole	Mazurais *et al.* (2014)
*MRPL19*	Downregulated in hypoxic conditions (in killifish)	Flight *et al.* (2011)
*OXNAD1*	Involved in hypoxia response; necessary for hypoxia cell survival	Jensen *et al.* (2011)
*PHKB*	Upregulated in response to hypoxia in rainbow trout	Léger *et al.* (2021)
*PNPT1*	Downregulated following oxidative stress in obscure pufferfish	Wen *et al.* (2019)
*PSMA6*	Functionally enriched in American alligator cardiac tissue following hypoxia; downregulated under hypoxic conditions in threespine stickleback	Leveelahti *et al.* (2011); Alderman *et al.* (2019)
*RNFT1*	ER-associated degradation pathway linked to hypoxic and heat stress in hard clams	Hu *et al.* (2022)
*RUVBL1*	Linked to CNVs in Chinese indigenous cattle, associated with lower copy numbers in high-altitude populations, linked to hypoxic stress	Zhang *et al.* (2020)
*TMEM150C*	Correlated with higher lung diffusing capacity and gene expression in rats raised at low oxygen levels (simulating high-elevation environment)	Krishnan *et al.* (2020)
*TMTC2*	Positively selected and linked to hypoxic adaptation in Chinese goats	Tian *et al.* (2021)
*TRIM35*	Downregulated under hypoxic conditions in adult channel catfish	Yang *et al.* (2018)
*UTP18*	Upregulated under anoxic conditions in yeast	Lee *et al.* (2016)
*ZPLD1*	Differentially expressed under varying hypoxic conditions in sea cucumber	Zhang *et al.* (2018)
**UV exposure**		
*DGCR8*	Phosphorylated after UV exposure, critical for cellular resistance to UV, repair of DNA damage, recovery of RNA synthesis in both mice and humans	Calses *et al.* (2017)
*DNA2*	May be linked to DNA repair following UV damage	Kciuk *et al.* (2020)
LOC101527142 (*CRHBP*)	Increased expression after exposure to UV in Chinese honeybees	Liu *et al.* (2011)
*MLH1*	Involved with mammalian DNA mismatch repair pathway, could be responding to ionizing radiation	Martin *et al.* (2010)
*RFC4*	Critical for DNA repair following UV damage; localizes to UV-stalled replication forks, leads to increased postreplication repair	Pathania *et al.* (2011)
*SERPINB2*	Significantly increased expression following UV irradiation, related to DNA repair (keratinocytes)	Majoros *et al.* (2019)
*TELO2*	Involved in cellular resistance to DNA damage, linked to an adaptive microsatellite in *Ciona robusta*	Chen *et al.* (2018)
**High elevation**		
*ADAL*	Environment-specific alleles associated with elevation in human populations	Gloria-Bottini *et al.* (2000)
*GPR83*	linked to an SNP outlier related to elevation in deer mice	Schweizer *et al.* (2021)
LOC101526896 (*UQCRC2*)	Over-expressed in yaks relative to cattle; improved energy metabolism due to high-altitude adaptation	Wen *et al.* (2019)
*RUVBL1*	Linked to CNVs in Chinese indigenous cattle, associated with lower copy numbers in high-altitude populations	Zhang *et al.* (2020)
*TMTC2*	Positively selected for in Tibetan sheep, possibly linked to high-altitude adaptation	Yang *et al.* (2016)
*UTP18*	Linked to adaptation to high-elevation environments in African indigenous chickens	Gheyas *et al.* (2021)

PSGs were identified from single-copy orthologs shared among 9 mammalian species using a branch-site model as implemented in CodeML in PAML *v*4.9 (Yang 2007).

## Discussion

### Life at high elevations

High-elevation environments are characterized by several extreme conditions including lower atmospheric oxygen content (hypoxia), reduced temperatures, and higher levels of UV radiation relative to what is found at lower elevations (Sun *et al.* 2018); each of these present significant challenges to the occupying fauna. Hypoxia reduces oxygen supply to tissues, which can limit aerobic metabolism (Cheviron and Brumfield 2012). A lowered metabolic rate can also lead to a reduction in thermogenesis in endotherms, making these species more susceptible to colder temperatures (Cheviron and Brumfield 2012). High levels of UV radiation pose a different challenge, particularly UV-B radiation, as this radiation can cause increased DNA damage (Wang *et al.* 2014). Species must adapt to these unique challenges in order to survive life at high elevations, often at a genomic level (Cheviron and Brumfield 2012; Qu *et al.* 2020). These genomic adaptations can affect genes responsible for oxygen transport (e.g. heme binding), energy metabolism, and DNA repair (Li *et al.* 2018; Sun *et al.* 2018). Putative adaptations to hypoxia have been found in several species of pika, including the American pika (Lemay *et al.* 2013; Waterhouse *et al.* 2018), Daurian pika (Solari and Hadly 2020), and plateau pika (Zhao *et al.* 2004; Li *et al.* 2009). Wang *et al.* (2020) identified putative adaptations associated with cold-tolerance across all extant pikas (*Ochotona* spp.) and hypothesized these occurred early in their evolutionary history (Wang *et al.* 2020). While these studies provide some insights into adaptation to high-elevation environments for some members of Ochotonidae, our study is the first to examine these questions on a whole-genome scale for the American pika, specifically.

### High-elevation adaptation in the American pika

As a first step for our comparative genomic investigation of American pika adaption to high-elevation environments, we reconstructed a phylogeny and estimated divergence times between our focal species and 8 other mammalian species strategically targeted based on the availability of an annotated reference genome, phylogenetic relatedness inferred from previous studies, and (dis)similarity in ecology. Our recovered topology had high nodal support and was consistent with previously estimated relationships (Gupta and Suggett 2022). In addition, our divergence time estimates largely matched previous fossil and molecular estimates for all nodes in the tree (Giboulet *et al.* 1997; Harrison *et al.* 2003; Benton and Donoghue 2007; Montgelard *et al.* 2008; Meredith *et al.* 2011; Voloch *et al.* 2013; Wang *et al.* 2020). Not only are these results consistent with prior hypotheses regarding phylogenetic relationships, but they also provide validation that the orthologs used in these analyses were appropriate for estimating evolutionary relationships among these species.

Using these identified orthologs, we found extensive evidence for putative high-elevation adaptation in the American pika genome. Both the pika-specific genes and expanded gene families showed functional enrichment in GO categories related to the oxidation-reduction process, including cytochrome-c oxidase activity (GO:0004129), heme-copper terminal oxidase activity (GO:0015002), hydrogen ion transmembrane transporter activity (GO:0015078), and 3 terms associated with oxidoreductase activity (GO:0016675, GO:0016676, and GO:0016491; Fig. 1; see Supplementary Tables 4 and 6). Cytochrome-c oxidase activity and child terms of both hydrogen ion transmembrane activity and oxidoreductase activity have been linked to hypoxia adaptation in high-elevation domestic yaks (*B. grunniens*; Qiu *et al.* 2012) and Tibetan snakes (*Thermophis* spp.; Li *et al.* 2018) located on the QTP. Interestingly, the expanded gene family with oxidoreductase activity contained 6 genes annotated to aflatoxin B1 aldehyde reductase genes, which are predicted to be involved in cellular aldehyde metabolic processes. One of these genes, *AKR7A3*, has also been associated with temperature and hypoxia in European flounder (Pédron *et al.* 2017), and both *AKR7A2* and *AKR7A3* have been linked to an SNP outlier among high-elevation adapted human populations (Foll *et al.* 2014). We also found evidence for 23 PSGs with various links to survival in hypoxic conditions (Table 1). These genes contribute to the oxidation-reduction process (e.g. *OXNAD1*), are localized to the mitochondria (e.g. *AASS*, *BCKDHA*, *MRPL19*, *PNPT1*), or are part of enzymatic and metabolic pathways (e.g. *AASS*, *ADAL*, *BCKDHA*, *CUL1*, *GBE1*, *MAT2B*, *PHKB*, *PNPT1*, *PSMA6*), which are all involved in cellular adaptation to hypoxia (Lee *et al.* 2020). Two of these PSGs, *OXNAD1* and *PHKB*, were also linked to outlier SNPs detected in a range-wide analysis of adaptation in the American pika (Schmidt *et al.* unpublished) and could serve as interesting targets for future study. Furthermore, we saw functional enrichment of numerous GO terms related to mitochondrial function and development, as well as metabolic activity (Fig. 1; see Supplementary Tables 4 and 6), providing further evidence for hypoxic adaptation in the American pika. Two of these terms, mitochondrion (GO:0005739) and mitochondrial inner membrane (GO:0005743), have been previously linked to upregulated genes in high-elevation populations of Himalayan pikas (*Ochotona royeli*; Solari *et al.* 2018).

Many of the putative hypoxia adaptations we found could also be linked to cold tolerance. American pikas have a high basal metabolic rate relative to their body size, leading to a higher than expected mean body temperature of ∼40°C (MacArthur and Wang 1974). The adaptations which increase metabolic output and/or mitochondrial function could have evolved in the American pika to combat the cold, ambient temperatures (Lemay *et al.* 2013; Rankin *et al.* 2017; Waterhouse *et al.* 2018; Wang *et al.* 2020). In addition to the above genes, we also found 16 PSGs with putative cold adaptations including those with metabolic functions (e.g. *PLA1A*, *SUGT1*, *TREH*) or localized to the mitochondria (e.g. LOC101526896, *NRDC*). Of particular interest, *NRDC* is predicted to be involved in membrane proteolysis and regulation of endopeptidase activity within mitochondria. Experiments with knockout mice found that *NRDC*-deficient lines were prone to hypothermia and severe cold intolerance, suggesting that *NRDC* plays an important role in thermogenesis and body temperature homeostasis (Hiraoka *et al.* 2014). This gene, in particular, represents a promising target for future studies to investigate the effects of various mutations on thermogenesis and cold tolerance in the American pika and other high-elevation mammals.

We further found evidence for adaptation to increased UV exposure in the American pika genome. We identified 16 enriched GO terms in the pika-specific dataset involved in DNA repair, as well as an additional term related to DNA DSB processing (Fig. 1; see Supplementary Table 4). Of the genes annotated with these terms, *UBE2V2* plays a role in DNA repair, particularly with respect to DSBs, such as those caused by UV radiation (Hofmann and Pickart 1999; David *et al.* 2010). This enzyme forms a heterodimer with *UBE2N* to catalyze the synthesis of “Lys-63”-linked polyubiquitin chains, which are necessary for error-free DNA repair (Hofmann and Pickart 1999; David *et al.* 2010). We also detected several promising PSGs related to DNA repair and cellular resistance to UV damage (Table 1). Several of these genes, namely *DNA2*, *MLH1*, and *RFC4*, appear to play a critical role in DNA repair following UV-induced DNA damage (Martin *et al.* 2010; Pathania *et al.* 2011; Kciuk *et al.* 2020). *DNA2* is involved in the 5′ resection of DNA during DSB repair (Kciuk *et al.* 2020; Zhao *et al.* 2020), while *MLH1* is part of the MutL alpha complex, a key component of the DNA mismatch repair system (Martin *et al.* 2010). Following UV damage, *RFC4* localizes to UV-stalled replication forks and contributes to checkpoint activation, leading to an increase in postreplication repair (Pathania *et al.* 2011). Other PSGs we found are involved in cellular resistance to UV damage. Phosphorylation of *DGCR8* following UV exposure appears to be critical for cellular resistance to UV, as well as for recovery of RNA synthesis in both mice and humans (Calses *et al.* 2017). *TELO2* acts as a regulator of the DNA damage response and is heavily involved in cellular resistance to both ionizing and UV radiation (Hurov *et al.* 2010; Chen *et al.* 2018). Collectively, these genes provide promising evidence for adaptation to increased UV exposure in the American pika.

### Other environmentally associated adaptations and considerations

While this study focused on putative high-elevation adaptations, we did find evidence for additional adaptations related to environment. We found significant enrichment for olfactory receptor activity among both pika-specific genes as well as expanded gene families in the American pika (see Supplementary Tables 4 and 6). American pikas have 2 types of foraging behavior: grazing (direct consumption) and haying (caching plants for an over-winter food supply; Huntly *et al.* 1986); in addition, American pikas appear to cache higher-quality vegetation rather than the most commonly available (Smith and Erb 2013). Enhanced olfaction could potentially aid American pikas in selecting the appropriate quality of food to best survive the winter months. Related to olfaction, we also saw enrichment of pheromone receptor activity among expanded gene families (see Supplementary Table 6). Unlike other lagomorphs, both male and female American pikas remain territorial year-round (Boonstra *et al.* 2022) and are known to use cheek glands to scent mark rocks around their territory to ward off conspecifics (Meaney 1986). This association between olfactory receptor activity and territorial behavior in American pikas represents a promising avenue for future inquiry.

It is important to note that the available American pika genome was constructed from a single individual sampled within Beaverhead-Deerlodge National Forest in southwestern Montana, USA at an elevation of ∼2,770 m above sea level. This site is centrally located within the Northern Rocky Mountains lineage of the American pika, which is the largest in terms of area (see Galbreath *et al.* 2009). The fact that the genome was constructed from a single individual from a single location could mean that observed variation may not be representative of the entire species. However, as we focused this study on coding regions, intraspecific variation should be less pronounced given evolutionary constraints including slower mutation rates. In addition, this genome should be appropriate for detecting high-elevation adaptations within the American pika given the sampling location approaches the upper extent of their contemporary elevational range (Smith and Weston 1990).

### Conclusions

Here, we identified and characterized putative adaptation in the American pika genome. We found support across multiple analyses for functional enrichment in categories related to hypoxia, cold tolerance, and DNA repair, and identified numerous PSGs with links to high-elevation adaptation. Although these results do not constitute direct evidence for environmental adaptation, they provide important targets for future studies within the American pika and across other mammalian species. These investigations could include examination of gene expression along elevational gradients, molecular assays in which functional responses are measured, or even correlative approaches of genetic differentiation across varying environmental conditions. Recommended targets include *OXNAD1*, *NRDC*, and those genes which are critical in DNA repair, as we found the strongest support for these regions. Altogether, this work provides the first whole-genome examination of high-elevation adaptation in American pikas and will serve as an important reference for future studies related to environmental adaptation and climate change.

## Supplementary Material

jkac241_Supplementary_Tables

jkac241_Supplemental_Material_Legends

jkac241_Supplementary_Figure_S1

## Data Availability

All genome assemblies and associated data are publicly available in the NCBI database with accession information in Supplementary Table 1 in the Supplementary Tables file. All custom scripts used in this article are available at https://github.com/bsjodin. Supplemental material is available at G3 online.
