# Supplementary material for: Comparative genomics reveals putative evidence for high-elevation adaptation in the American pika (Ochotona princeps)
Source: G3 (Bethesda). 2022 Sep 10;12(11):jkac241. doi: 10.1093/g3journal/jkac241 (PMC9635661; doi:10.1093/g3journal/jkac241)
Supplement: jkac241_Supplemental_Material_Legends [file jkac241_supplemental_material_legends.docx]

**SUPPLEMENTAL LEGEND**

Figure S1. Consensus tree for nine mammalian genomes. The phylogeny was estimated from concatenated orthologous gene alignments (4,777 genes; total length = 739,038 bases) under the GTR+I+Γ model as implemented in MrBayes *v*3.2.6 (Ronquist *et al.* 2012) across five independent MCMC runs of five million generations each.
