# Supplementary figures and images for: Comparative genomics reveals putative evidence for high-elevation adaptation in the American pika (Ochotona princeps)
Source: G3 (Bethesda). 2022 Sep 10;12(11):jkac241. doi: 10.1093/g3journal/jkac241 (PMC9635661; doi:10.1093/g3journal/jkac241)

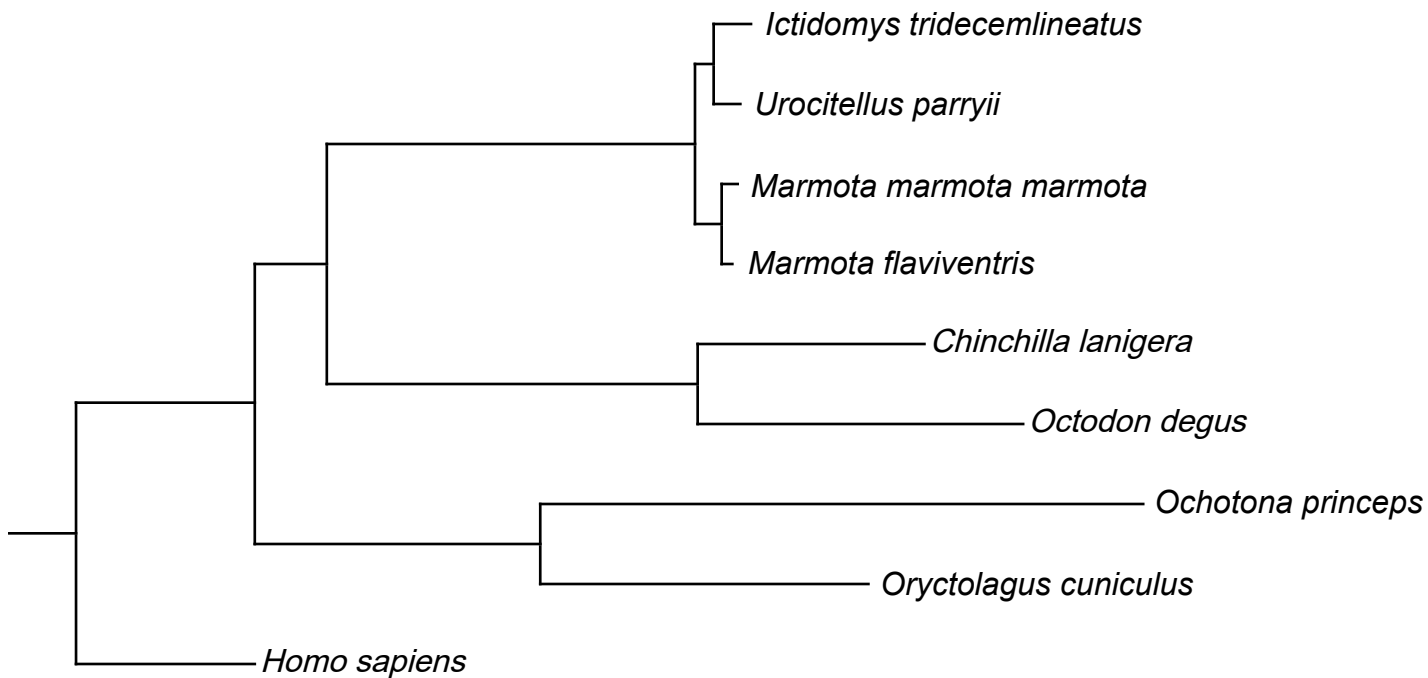

0.10

Supplement: jkac241_Supplementary_Figure_S1 [file jkac241_supplementary_figure_s1.pdf]
